# 3D structural modeling of lower cretaceous Alam El Bueib reservoir in Menes field, Shushan Basin, north Western Desert, Egypt

**DOI:** 10.1038/s41598-025-07375-x

**Published:** 2025-06-27

**Authors:** Hany Samy Ibrahim, Walid M. Mabrouk, Ahmed M. Metwally

**Affiliations:** Geophysics Department, Faculty of Science, Cairo University, Giza, 12613 Egypt

**Keywords:** 3D structure modeling, Shushan Basin, Menes field, Lower cretaceous, Geology, Geophysics

## Abstract

This study is focused on the Menes oil field, located on the western flank of Shushan Basin in Egypt’s northern Western Desert (NWD). The primary oil-bearing reservoir in this area is the Lower Cretaceous Alam El Bueib (AEB) Formation (Fm), that extends through the Barremian to Aptian stages. This formation is characterized by thick, massive, argillaceous, and calcareous sandstones interbedded with shale and carbonate layers. 2D seismic profiles are interpreted to delineate the structural features of the subsurface. The well to seismic tie via synthetic seismograms and check-shot data are utilized for mapping the formation tops of Alamein dolomite, as well as the AEB units (1, 3 A, 3 C, and 3D), and the Paleozoic strata. Electrical wireline logs from four wells in Menes oil field were analyzed to estimate key petrophysical parameters, including porosity and hydrocarbon saturation for reservoir characterization. Finally 3D structural model was developed to enhance subsurface visualization, enabling a more precise characterization of the AEB reservoirs. This model also aims to reduce exploration risks and improve field development strategies in the study area. These findings provide crucial insights into the subsurface characteristics and hydrocarbon prospects of this formation, offering valuable information that can inform strategic decision-making in both exploration and production activities within Shushan basin. The comprehensive understanding gained from these results serves as a key contribution to optimizing future exploration efforts and enhancing the development of hydrocarbon resources in the near by regions.

## Introduction

The northern part of Egypt’s Western Desert is considered one of the most significant hydrocarbon provinces, with effective exploration and development of fields^1^. It has extensional coastal rift basins like Matruh-Shushan, Alamein, and Abu Gharadig Basins^2,3^. These basins are recognized by several types of hydrocarbon traps, primarily associated to the Late Cretaceous tectonics of the Syrian Arc^4,5^. These traps typically include three- and four-way closures, trap doors, horsts, and block faults, with trends oriented from North West to South East (NW-SE) and North East to South West (NE-SW)^6–11^.

**Fig. 1:**
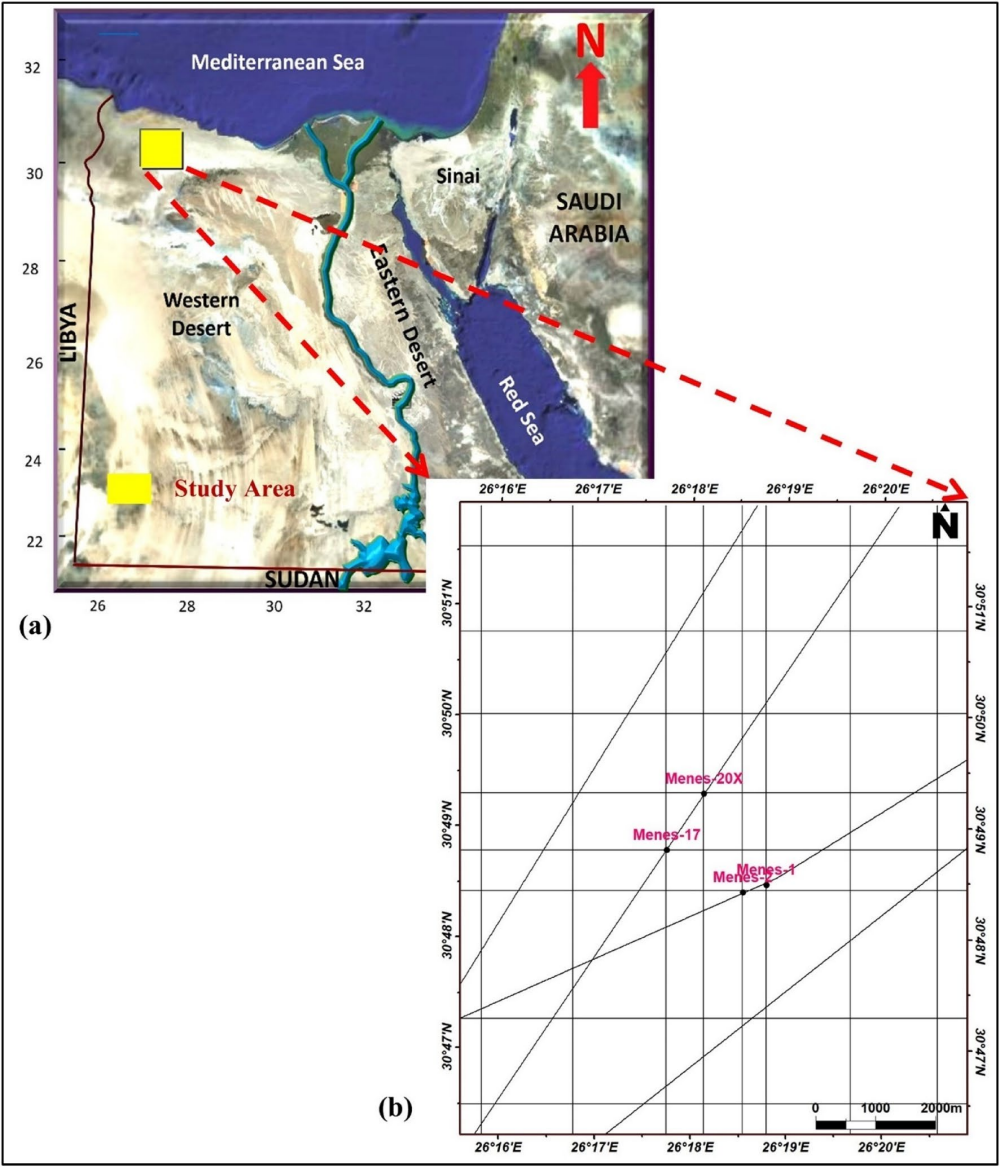
(**a**) Egypt’s geographic map revealing location of the study area. (**b**) Base map showing the available seismic lines and well locations.

**Fig. 2:**
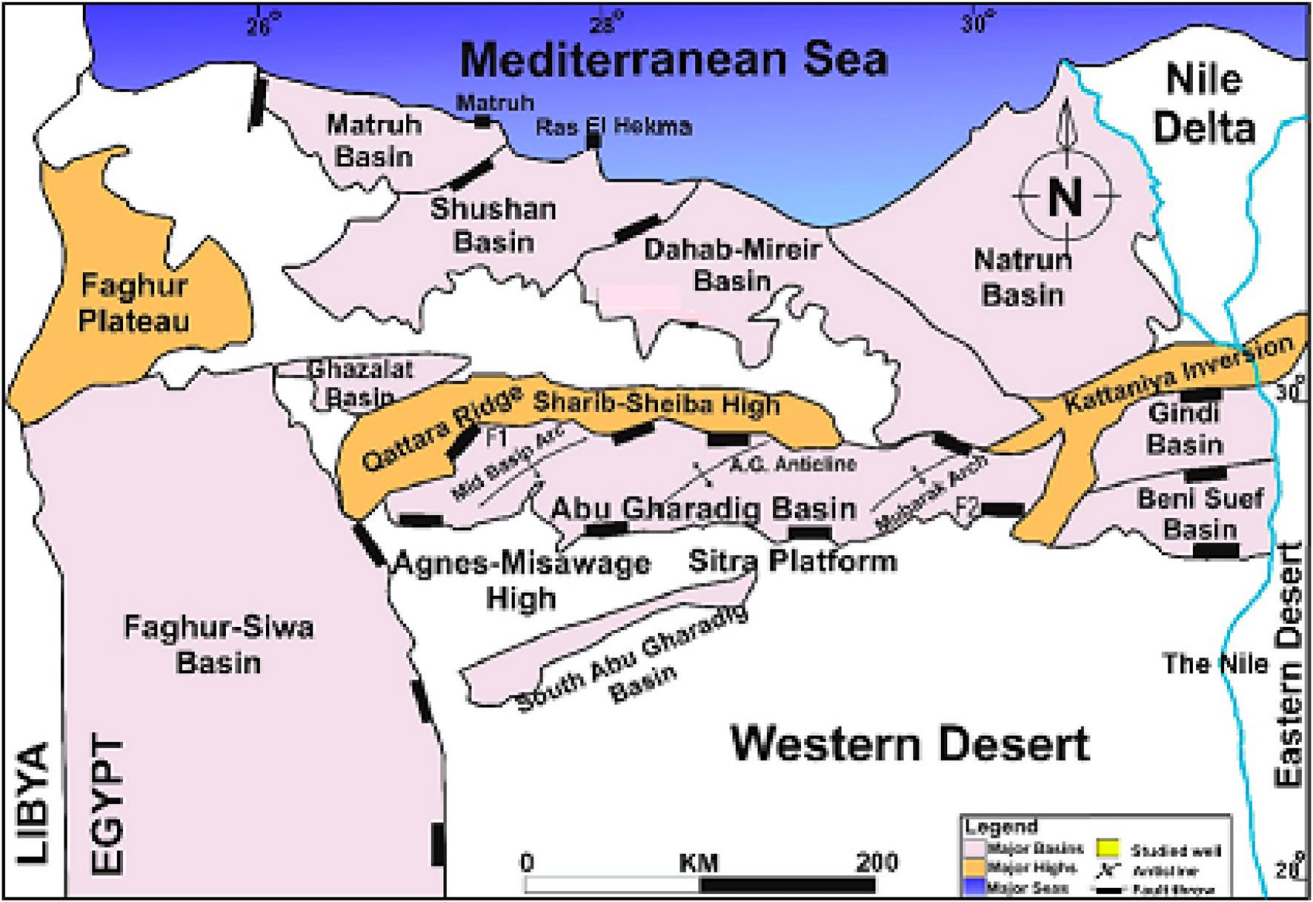
Main structural features of the north Western Desert, modified after^1^.

The Menes Field is situated in the Khalda concession in the western part of the Shushan Basin, approximately between 30°46’30"N, 30°51’30"N latitude, and 26°15’30"E, 26°20’30"E longitude (Fig. 1a & b). This basin is bounded by the Faghour Plateau to the west, the Faghur-Siwa basin from the Southwest, and the Qattara Ridge to the South (Fig. 2).

In general, the Khattaba Formation is considered the main possible source rock in the Shushan Basin, which extends up to 7650 m at the basin depocenter^12,13^.

Additionally, the Alam-El Bueib Formation is the main reservoir, consisting mainly of sandstones with siltstones and a few streaks of shales^14^. Lithology is split into six units settled from base to top Alam El Bueib (6, 5, 4, 3, 2, and 1). The Alam El Bueib-3 unit is also split into six subunits (A, C, D, E, F, and G).

This research primarily aims to build a three-dimensional structural geological model of the Menes Field, located in the Shushan Basin of Egypt’s Western Desert. The primary objective of this model is to improve the understanding of the subsurface structural framework, especially focusing on the configuration and geometry of lower cretaceous (AEB) formations. The seismic interpretations and well data were integrated, enabling the 3D model to: (1) provide an accurate image of subsurface structures in the Menes Field through the identification of reservoir boundaries and fault systems; (2) enhance the characterization of the Lower Cretaceous AEB reservoirs by evaluating rock properties and fluid distributions; and (3) Suggest new prospective drilling locations, additionally minimizing risks associated with the planning and drilling of future wells.

## Geological setting

Four main cycles are found in the Mesozoic and Cenozoic sections, each ending with a marine transgression as explained by Sultan and Halim^15^. The lower cycle includes the Lower Jurassic non-marine clastics of the Ras Qattara Formation and the Middle Jurassic Khatatba Formation. Also, during the Late Jurassic, shallow marine carbonates were deposited in the Masajid Formation, showing the peak of the marine transgression in the Jurassic period. On local highs, the Masajid Formation is either eroded or not deposited.

Throughout the Early Cretaceous, the second cycle began with the deposition of shallow marine clastics from the Alam El Bueib Formation (units 6 and 5). This was followed by the deposition of marine shale (unit AEB-4) and large sand bodies, which were separated by marine shale intrusions (unit AEB-3). Shale, sandstone, and marine shelf carbonates (units 2 and 1) alternately cover these sand bodies, followed by the Alamein dolomite. The cycle ended with a thin shale unit (Dahab shale). A significant unconformity separates the Masajid and Alam El Bueib Formations, while another unconformity separates the Dahab Shale and Kharita Formations. On local highs, the Alamein Dolomite is either eroded or not deposited.

The third cycle covers the Early Tertiary into the Middle Albian. It starts with the regressive period noted by the continental and shoreline sands of Kharita Formation. In the early Cenomanian, the Bahariya Formation was deposited on a shallow marine shelf, followed by the deposition of the Abu Roash (G) unit during the Late Cenomanian, showing an increase in sea level.

During the Turonian, the members of Abu Roash (F-A), mainly consisting of carbonates, were deposited due to a widespread transgression of sea. During Senonian, the Khoman Chalk Formation was deposited. The third cycle ends with unconformity.

**Fig. 3:**
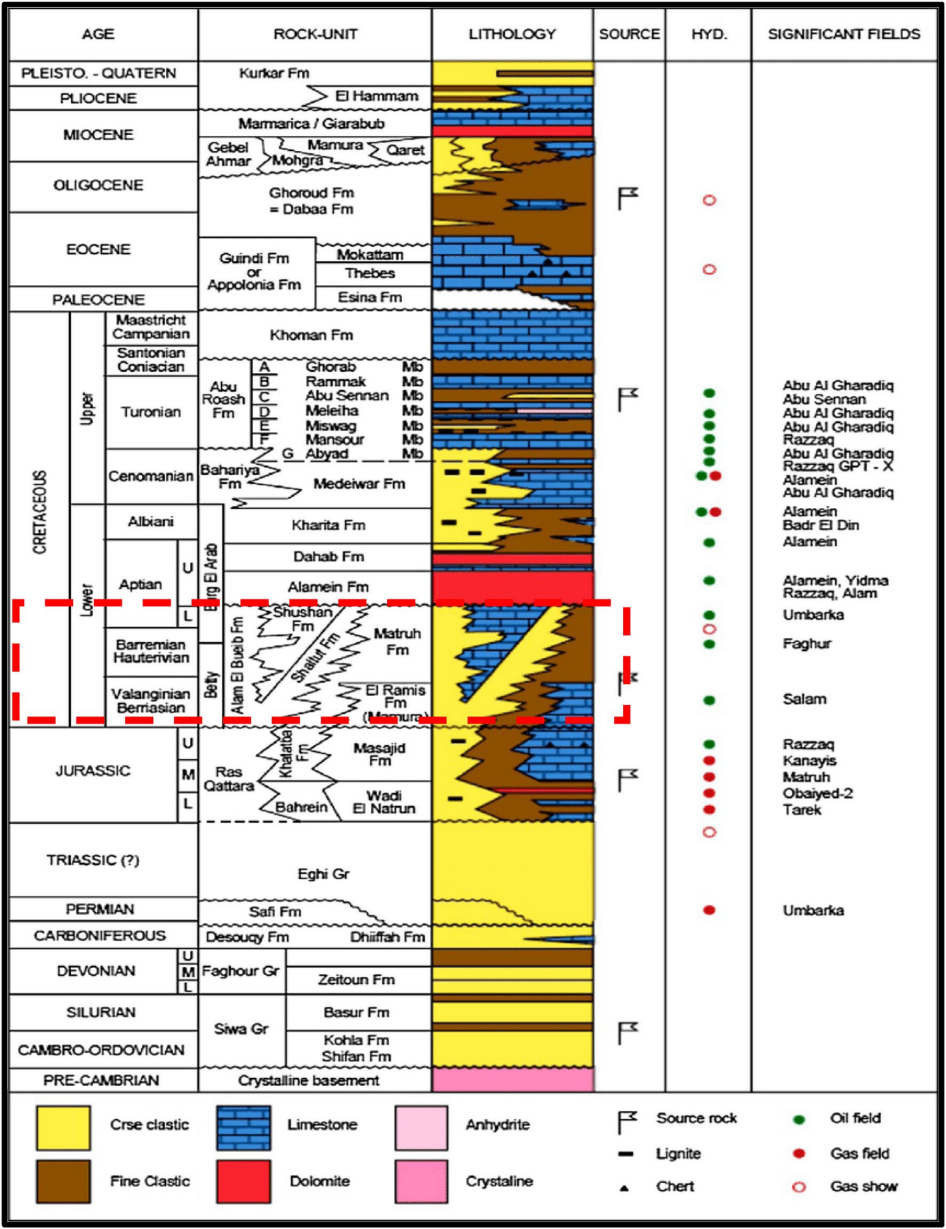
General stratigraphic column of Egypt’s north Western Desert, modified from^1,50^.

This is followed by the Eocene Apollonia Formation, marking the base of the fourth cycle including the Dabaa and Moghra’s marine clastics. Additionally, Marmarica Limestone was deposited as the cap rock of Moghra. On local highs and platforms, the Dabaa was not deposited^16^ (Fig. 3).

El Awdan et al.^17^ mentioned that the Shushan Basin was affected by the early Cretaceous and Jurassic extension, followed by Late Cretaceous and Early Tertiary inversion. The Alam El Bueib Formation is seen by high-quality reservoirs, marked by high porosity and excellent permeability^18^.

## Materials and methods

The current work is based on the Twenty 2D reflected seismic lines covering the study area in both dip and strike directions (Fig. 1b) and well-log data from four wells (Mnes-1X, Mnes-2, Mnes-17, and Mnes-20X). The logs have gamma rays, resistivity, neutron, density, sonic, photoelectric, and caliber logs.

**Fig. 4:**
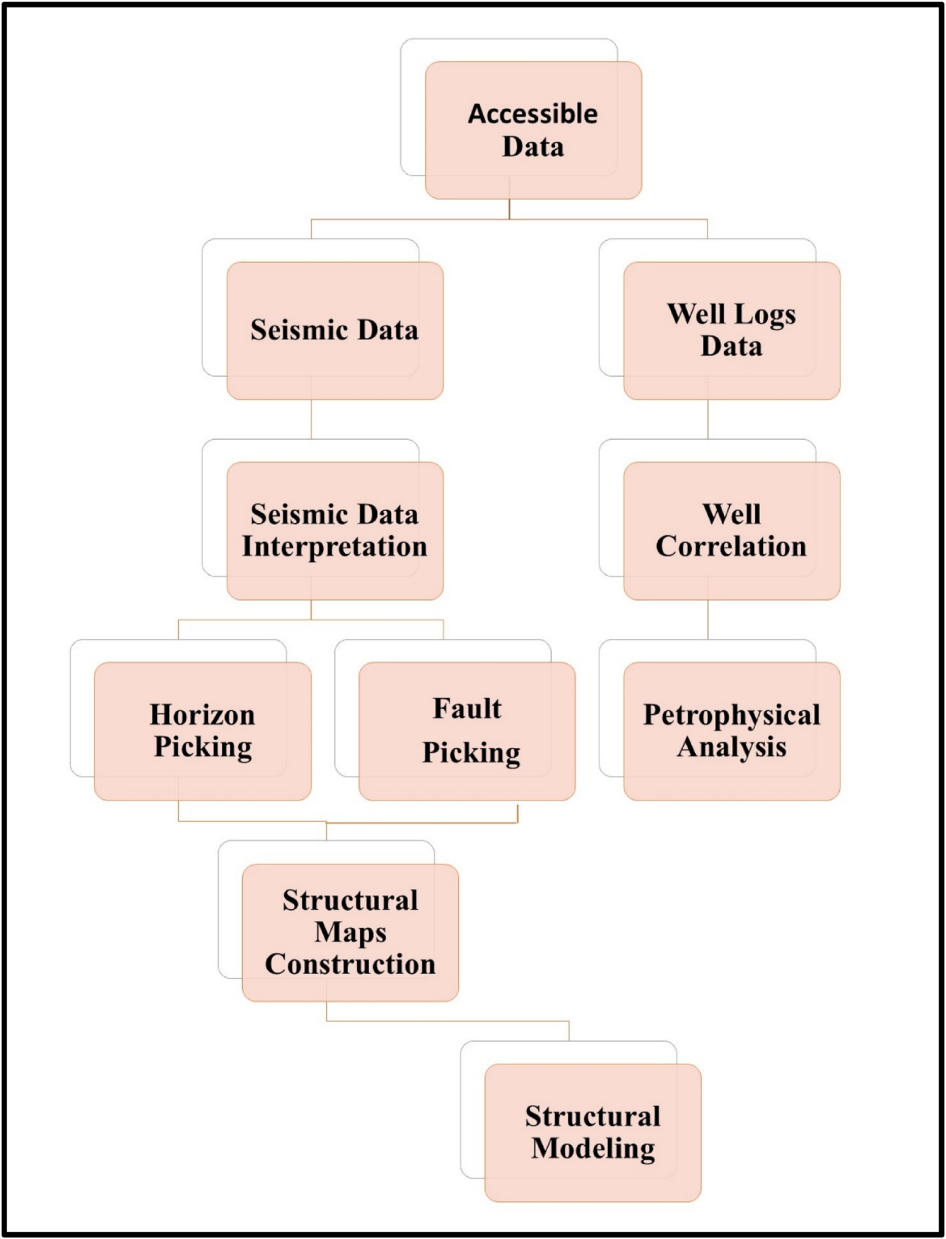
Displays the applied 3D structural modeling flowchart in this study.

The main goal of this research is to define the key geological horizons within the Alam El Bueib members and Alamein Formation in the study area, identify the affecting structural elements including faults, folds, and other geological features that influence the subsurface structures and build a 3D structural model of the lower Cretaceous Alam El Bueib reservoir. Figure 4 illustrates the flowchart for constructing the 3D structural model.

PetrelTM (version 2017.4) by Sschlumberger Inc was used for interpreting seismic sections, creating structure maps and building 3D models to understand the study area’s structural framework. The software of Interactive Petrophysics (IP), version 4.2, was used for Formation evaluation, petrophysical analysis and well correlation, estimating the porosity and fluid saturation of reservoirs.

## Results and discussion

### Seismic data interpretation

Seismic data interpretation is controlled by the data features and the applied structural or stratigraphic techniques^19–24^.

Twenty seismic lines were fully interpreted to identify the subsurface structure of the study area. The accessible borehole data and 2D seismic lines are tied together to identify the reflector boundaries of various zones.

### Synthetic seismogram generation

The importance of the synthetic seismogram lies in tying well data (in depth) to seismic data (in time), which enables recognition of seismic reflections that match geological formations^25^.

The sonic curve logged in the Menes-1X well was calibrated using a check-shot survey. The calibrated sonic log, together with the log of density and a statistical seismic wavelet, was then used to generate the synthetic seismogram by convolving the reflection coefficient log with the specified wavelet using Petrel™ (version 2017.4)^26^.

**Fig. 5:**
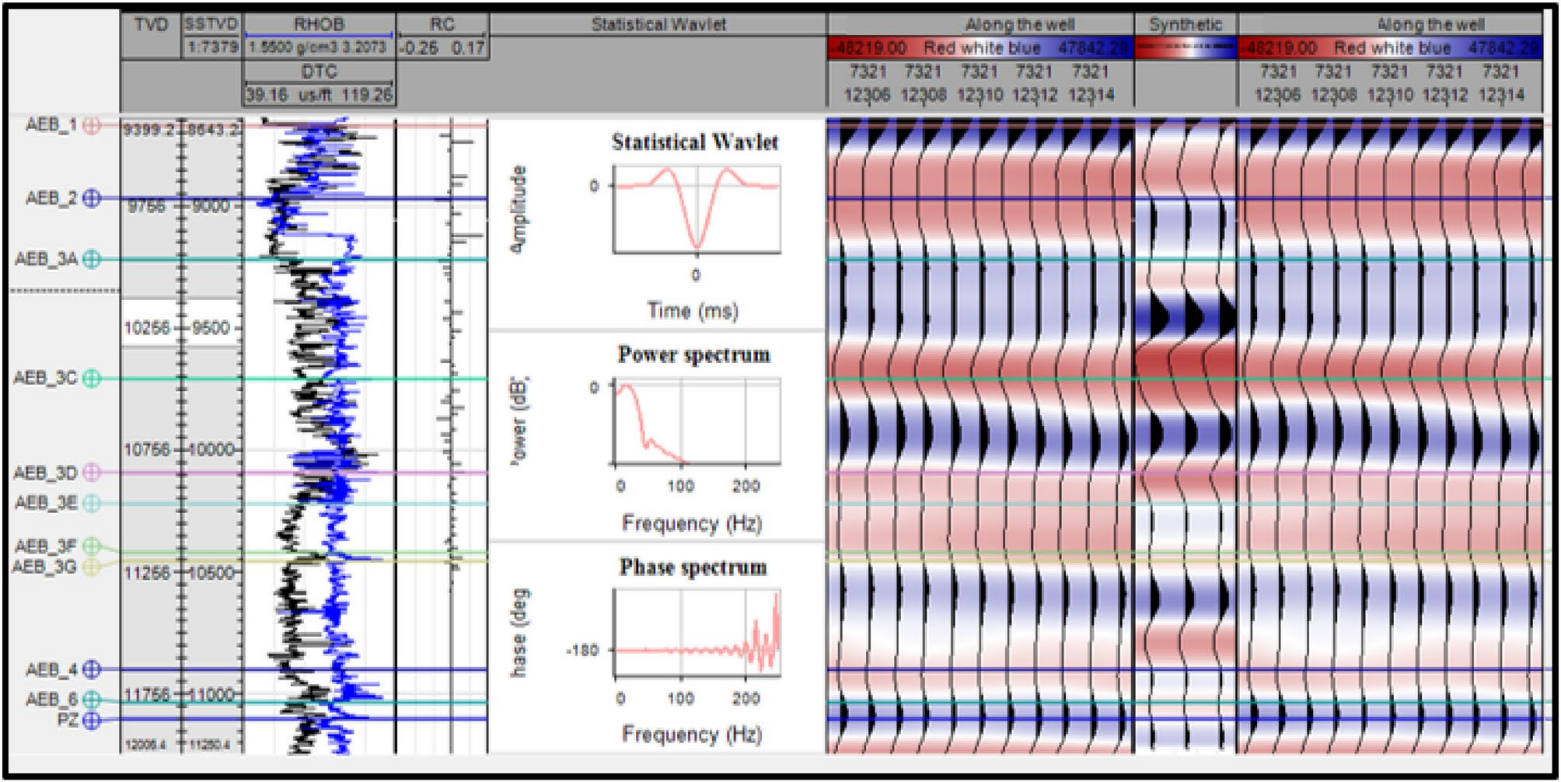
A synthetic seismogram for Well Menes-1X, created to correlate data of well log with the seismic section.

Figure 5 illustrates the correlation of the seismic cross sections with synthetic seismogram through well Menes-1X, marking the Alam EL Bueib members’ tops using the check-shot data.

### Horizons and faults picking

Seismic lines crossing wells are commonly used as the first step in horizon identification as they allow for the correlation between the stratigraphy and seismic reflections. Key horizons were picked to confirm local continuity and match properties. Horizons are usually interrupted by faults, making displacement^27,28^.

**Fig. 6:**
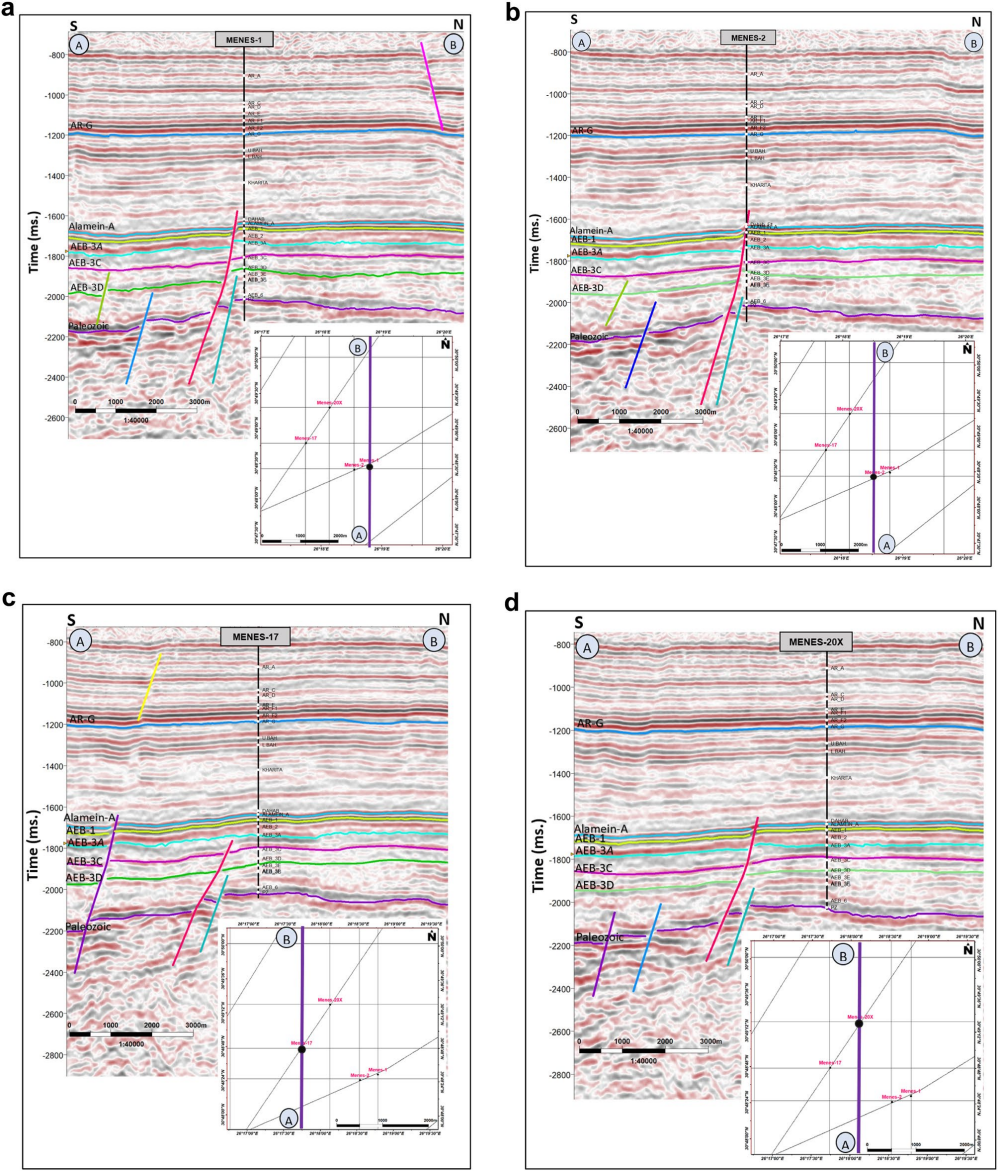
(**a**) Seismic section along line A-B passing through well Menes-1X, revealing the main interpreted stratigraphic horizons and normal faults. (**b**) Seismic section along line A-B passing through well Menes-2, revealing the main interpreted stratigraphic horizons and normal faults, where the well path intersects the major normal fault in Dahab formation with fault cut around 50 ft. (**c**) Seismic section along line A-B passing through well Menes-17, revealing the main interpreted stratigraphic horizons and normal faults. (**d**) Seismic section along line A-B passing through well Menes-20X, revealing the main interpreted stratigraphic horizons and normal faults

The top of the Alamein Formation, a continuous high-frequency seismic reflector, and the Paleozoic are identified across all seismic lines. Additionally, key reservoirs in the Alam El Bueib Formation were defined by picking the tops of units AEB-1, AEB-3 A, AEB-3 C, and AEB-3D, as shown in Fig. 6a-d. Furthermore, the well path of Menes-2 intersects a major normal fault within the Dahab Formation, resulting in a missing section of approximately 50 feet, as illustrated in Fig. 6b.

In general, these N-S seismic sections display set of normal faults, affecting the Lower Cretaceous sequences with a downthrown side to the south direction. In addition to that the Wells (Menes-1X, Menes-2, Menes-17, and Menes-20X) were drilled to test the upthrown side of the East -West normal fault targeting the AEB reservoirs.

Unfortunately, the carbonate section of Masajid Formation, which helps to define the base of the Alam El Bueib Formation, has been eroded in the study area.

### Structure maps construction

To explain the subsurface structure of the study area, the tops of six horizons (Alamein Dolomite, Alam El Bueib’s units (1, 3 A, 3 C, 3D) and Paleozoic were picked to create time structure contour maps then converted to depth domain using velocity maps.

The velocity model is applied to convert seismic data from time to depth domain. In this procedure, a seismic reference datum (SRD) is established at the beginning of the project. The applied method is the linear average velocity^29^:

1 $$\:V={V}_{0}+\text{K}\text{Z}$$

where $\:V$ refers to the velocity at the datum and $\:\text{Z}$ represents the vertical distance from the datum. $\:{V}_{0}$ is the velocity at Z = 0, and will, typically much lower than the velocities within the subsurface layers. $\:\text{K}$ is the gradient of velocity, ranging from 0 to − 0.2^30,31^.

**Fig. 7:**
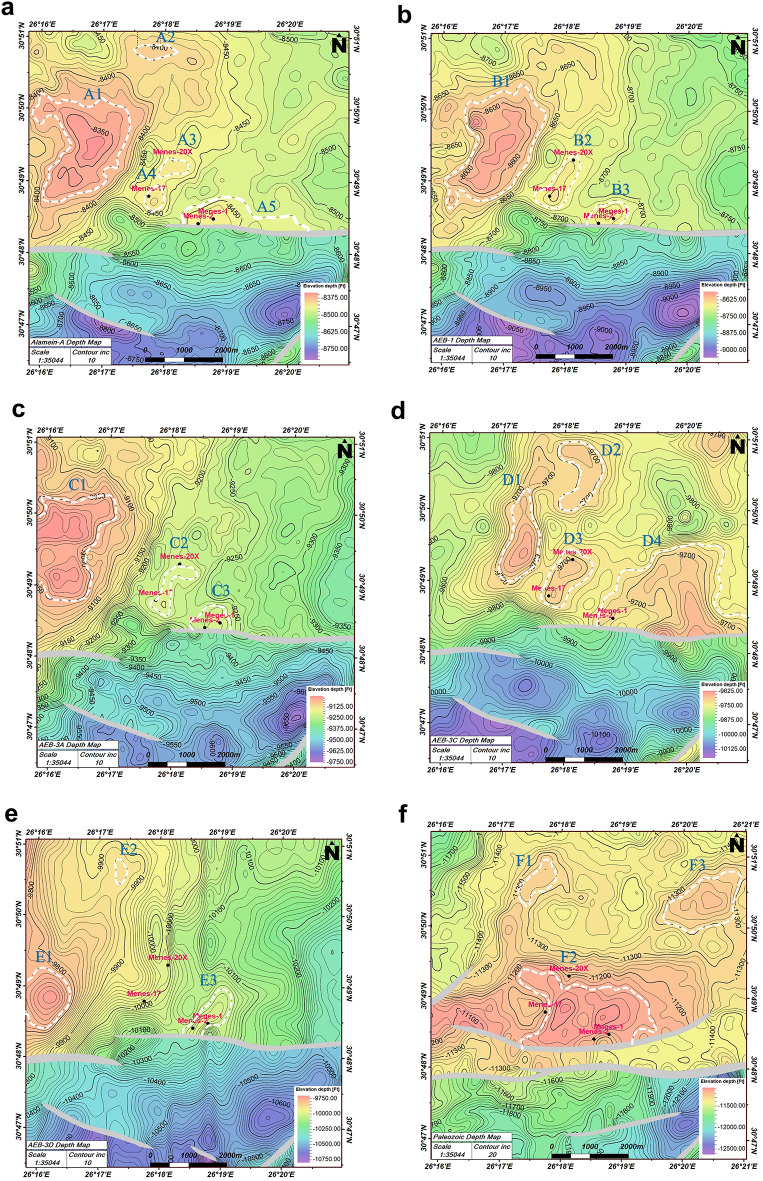
(**a**) Depth structure contour map of the Alamein-A unit with a contour interval of 10 feet. The map reveals the trend of the major faults, with the prospect locations (A1:A5) outlined by white dashed polygons, and the grey polygons indicating fault heaves. (**b**) Depth structure contour map of the AEB-1 unit with a contour interval of 10 feet. The map reveals the trend of the major faults, with the prospect locations (B1–B3) outlined by white dashed polygons, and the grey polygons representing fault heaves. (**c**) Depth structure contour map of the AEB-3A unit with a contour interval of 10 feet. The map reveals the trend of the major faults, with the prospect locations (C1–C3) outlined by white dashed polygons, and the grey polygons representing fault heaves. (**d**) Depth structure contour map of the AEB-3C unit with a contour interval of 10 feet. The map reveals the trend of the major faults, with the prospect locations (D1–D4) outlined by white dashed polygons, and the grey polygons representing fault heaves. (**e**) Depth structure contour map of the AEB-3D unit with a contour interval of 10 feet. The map reveals the trend of the major faults, with the prospect locations (E1–E3) outlined by white dashed polygons, and the grey polygons representing fault heaves. (**f**) Depth structure contour map of the Paleozoic unit with a contour interval of 20 feet. The map reveals the trend of the major faults, with the prospect locations (F1–F3) outlined by white dashed polygons, and the grey polygons representing fault heaves.

The depth structure contour maps (Fig. 7a-f) show patterns of normal faults with different trends East to West (E-W), North West to South East (NW-SE).) and North East to South West (NE-SW). The E-W fault forms a three-way dip closure structure. This structural pattern offers the probability of hydrocarbon presence in high areas, particularly where faults are sealed.

Table 1 highlights the summary of recognized probable prospects in the Menes field, sorted by structural type and target reservoir.

**Table 1:** Summary of recognized prospects in the Menes field, sorted by structural type and target reservoir.

No.	Prospect name	Prospect type	Target reservoir
1	A1	Four-way dip closure	Alamein-A
2	A2	Four-way dip closure	
3	A3	Four-way dip closure	
4	A4	Four-way dip closure	
5	A5	Up thrown three-way dip closure	
6	B1	Four-way dip closure	AEB-1
7	B2	Four-way dip closure	
8	B3	Three-way dip closure	
9	C1	Four-way dip closure	AEB-3 A
10	C2	Four-way dip closure	
11	C3	Up thrown three-way dip closure	
12	D1	Four-way dip closure	AEB-3 C
13	D2	Four-way dip closure	
14	D3	Four-way dip closure	
15	D4	Up thrown Three-way dip closure	
16	E1	Four-way dip closure	AEB-3D
17	E2	Four-way dip closure	
18	E3	Up thrown three-way dip closure	
19	F1	Four-way dip closure	Paleozoic
20	F2	Up thrown three-way dip closure	
21	F3	Four-way dip closure	

### Well correlation

Correlation between wells is essential to improve the quality of 3D geological model. In this study, correlation was used to show the differences in reservoir characteristics and thickness among the lower cretaceous reservoir’s geological units.

**Fig. 8:**
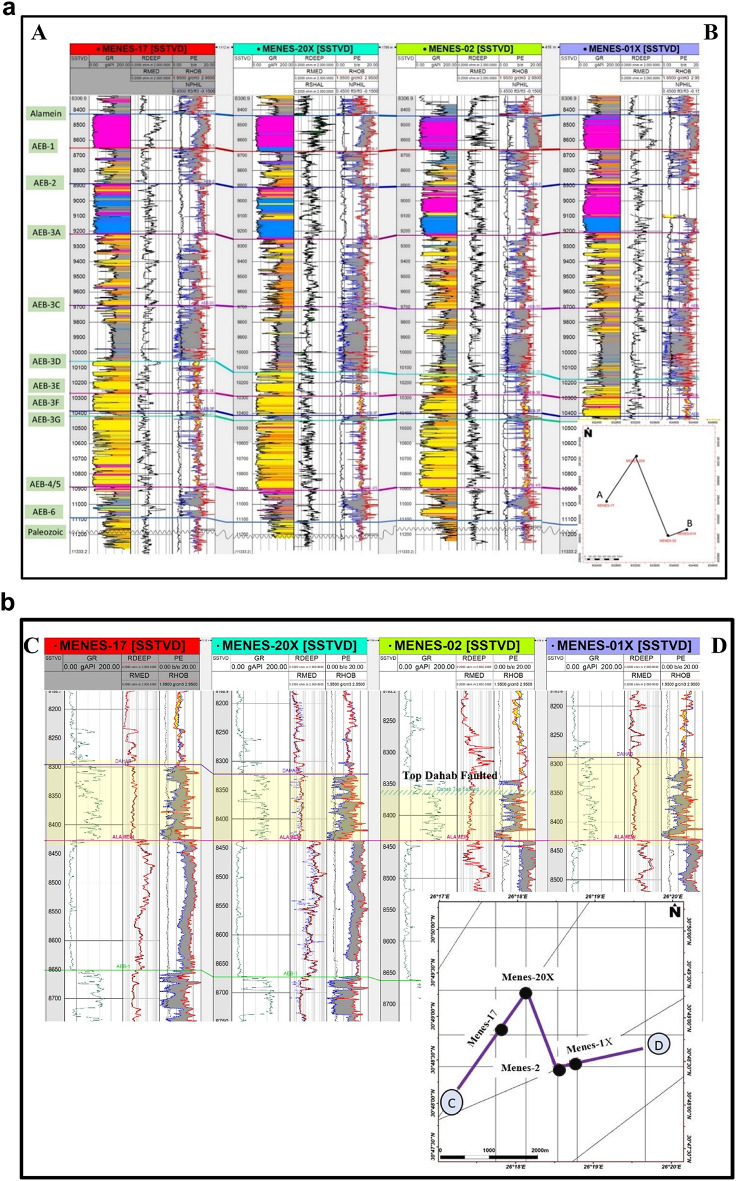
(**a**) A structural well correlation along the line (A-B) in the study area shows changes in lithology and well log responses. There is a slight increase in the thickness of the Alam El Buieb Formation toward the East. (**b**) A stratigraphic well correlation along line C-D for Menes wells flattened on Alamein Formation. The correlation reveals that there is approximately 50 feet missing section within the Dahab Formation due to a major normal fault intersected by the Menes-2 well.

Figure 8a represents a structural well correlation for the Menes-17, Menes-20X, Menes-2, and Menes-1X wells along line (A-B). This vertical section includes data for Gamma-ray, resistivity, neutron and density. The section reveals that the lithology is mostly clastic, consisting of sandstone, siltstone, and shale, with carbonate streaks present at the uppermost part of the Alam El Buieb Formation. Moreover, Menes-1X is considered slightly higher compared to the other wells. Additionally, there is a slight growth in the thickness of the Alam El Buieb Formation towards the East. This suggests favorable conditions for sandstone deposition, which could lead to higher-quality reservoir rocks.

**Fig. 9:**
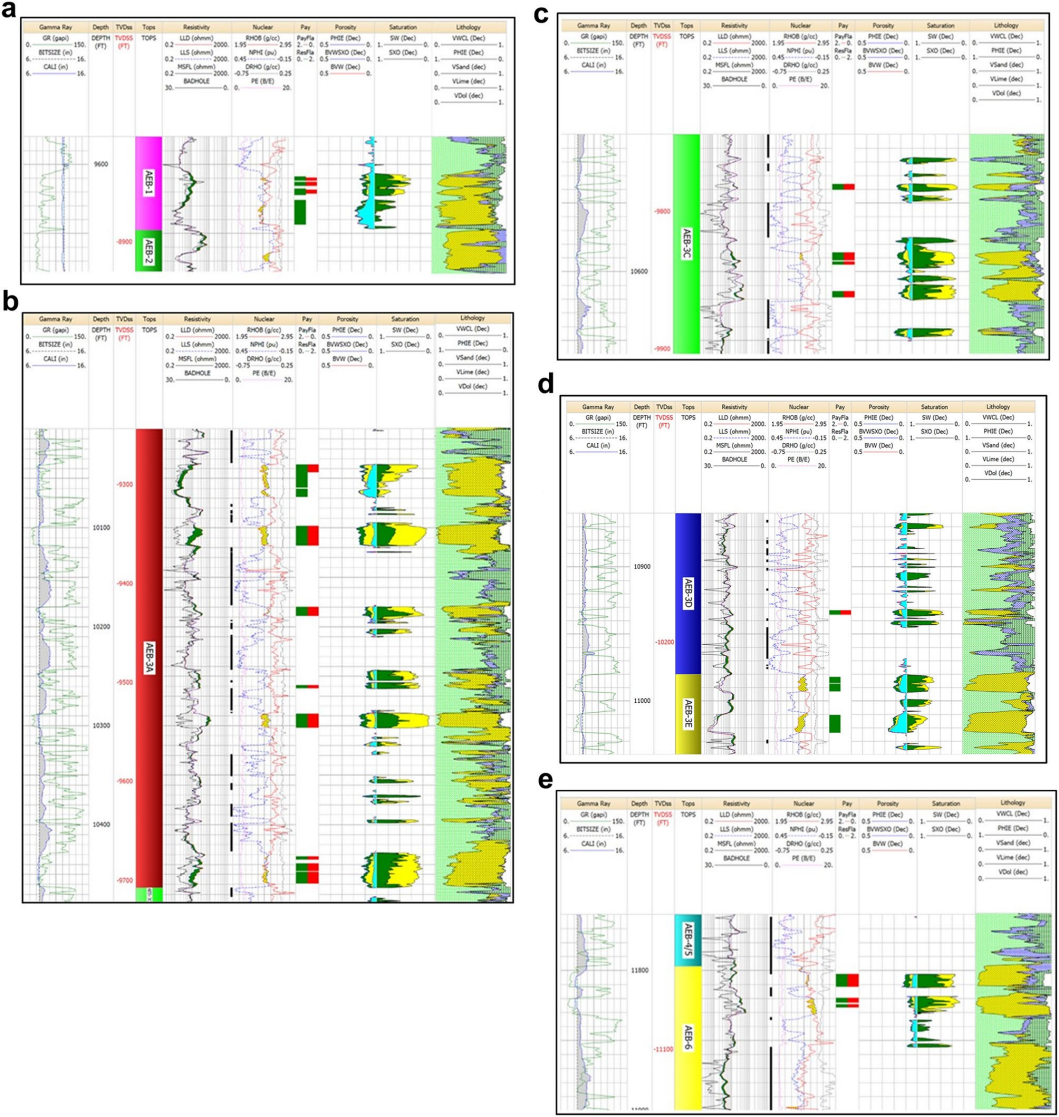
(**a**) Litho-saturation plot for Well Menes-1X in the AEB-1 Unit. The plot shows presence of 8 ft net pay, 11% average porosity and 42% average water saturation. (**b**) Litho-saturation plot for Well Menes-1X in the AEB-3A Unit. The plot shows presence of 75 ft net pay, 12% average porosity and 24% average water saturation. (**c**) Litho-saturation plot for Well Menes-1X in the AEB-3C Unit. The plot shows presence of 16 ft net pay, 11% average porosity and 27% average water saturation. (**d**) Litho-saturation plot for Well Menes-1X in the AEB-3D Unit. The plot shows presence of 3 ft net pay, 12% average porosity and 39% average water saturation. (**e**) Litho-Saturation Plot for Well Menes-1X in the AEB-6 Unit. The plot shows presence of 15 ft net pay, 10% average porosity and 38% average water saturation.

Figure 8b shows a stratigraphic well correlation along line C-D for the previously mentioned wells. The correlation implies that approximately 50 feet missing section within the Dahab Formation due to a major normal fault intersected by the Menes-2 well, as illustrated in the seismic section (Fig. 6b).

### Petrophysical analysis

The main purpose of petrophysical analysis is to retrieve useful data and information from the well logs^32^. The logs of gamma ray, resistivity, neutron and density were utilized to characterize the Alam El Buieb Formation and its fluid saturation. Well production in hydrocarbon-bearing reservoirs is greatly influenced by key petrophysical properties like lithology, porosity, and water saturation^7,33–36^.

Four wells’ electrical logs in Menes field were analyzed for the AEB Formation. The volume of shale was determined by analyzing the data of Gamma ray log, which helps to differentiate between shaly and clean formations and quantify the content of shale in the reservoir. The shale volume ($\:{V}_{sh}$) can be calculated using the following equation^37^:

2 $$\:{V}_{sh}=({GR}_{log}-\:{GR}_{min})/({GR}_{max}-{GR}_{min})$$

Where $\:{V}_{sh}$ and $\:{GR}_{log}$ refer to the shale volume and the gamma ray reading from logs, respectively. $\:{GR}_{max}$ and $\:{GR}_{min}$ represent the maximum and minimum gamma ray readings, respectively.

The total porosity was estimated using the density and neutron logs, according to the following formula^37^:

3 $$\:{\:\:\:\:\:\:\:\:\:\:\:\:\:\:\:\:\:\:\:\:\:\:\:\:\:\:\:{\varnothing}}_{T}=({{\varnothing}}_{N}+{{\varnothing}}_{D})/2$$

Where $\:{{\varnothing}}_{T}$ refers to the total porosity, while $\:{{\varnothing}}_{N}$ and $\:{{\varnothing}}_{D}$ represent the porosity derived from the neutron log, and the density log, respectively.

The effective porosity was estimated using the following formula^37,38^:

4 $$\:{{\varnothing}}_{eff}={{\varnothing}}_{T}-\left({V}_{sh}*\:{{\varnothing}}_{sh}\right)$$

Where $\:{{\varnothing}}_{eff}$ denotes the effective porosity and $\:{{\varnothing}}_{T}$ is the total porosity. Moreover, $\:{V}_{sh}$, $\:{{\varnothing}}_{sh}$ refer the shale volume, and shale porosity, respectively^27,36,39^.

Water saturation was estimated by the following Indonesian equation, which assesses the percentage of the pore volume occupied by water.

5 $$\:\frac{1}{\sqrt{{R}_{t}}}=\:\left[\sqrt{\frac{{{\varnothing}}^{m}}{a{R}_{w}}+}\frac{{V}_{cl}\left(\frac{1-{V}_{cl}}{2}\right)}{\sqrt{{R}_{cl}}}\right]{S}_{w}^{n}$$

Where$\:{\:S}_{w},\:\:n\:$denotes water saturation and exponent of saturation, respectively. While $\:{R}_{t}$ and $\:{V}_{cl}$ refer to true resistivity and the volume of clay or shale, respectively. Moreover, $\:{{\varnothing}}_{T}$ and $\:{{\varnothing}}_{sh}$ represent the total porosity and shale porosity, respectively. Also, a’, $\:{R}_{w}$ and $\:{R}_{cl}$ represent the tortuosity factor, resistivity of water formation, and the resistivity of clay or shale, respectively^35,40^.

The petrophysical analysis results are summarized in Table 2. The positive results were obtained for units AEB (1, 3A, 3C, 3D, and 6) in well Menes-1X, as shown in Fig. 9a-e. For the AEB-1 unit, the net pay is 8 feet, with an average porosity of 11% and an average water saturation of 43%. The AEB-3A unit has several zones of total net pay of 75 feet, with 12% average porosity and 24% average water saturation. The AEB-3C unit shows a net pay of 16 feet, 11% average porosity, and 27% average water saturation. For AEB-3D, the net pay is 3 feet, with 12% average porosity and 39% average water saturation. For AEB-6, the net pay is 15 feet, with 10% average porosity and 38% average water saturation.

**Table 2:** Summary of main petrophysical parameters (net pay thickness, average porosity, and average water saturation) for the AEB reservoir zones in the Menes field.

Well name	Zone	Net pay thickness (Ft.)	Average porosity (%)	Average water saturation (%)
Menes-1X	AEB-1	8	11	43
	AEB-3 A	75	12	24
	AEB-3 C	16	11	27
	AEB-3D	3	12	39
	AEB-6	15	10	38
Menes-2	AEB-1	14	11	38
	AEB-3 A	60	12	27
	AEB-3 C	32	13	23
	AEB-3D	14	10	20
	AEB-6	16	8	22
Menes-17	AEB-1	4	10	39
	AEB-3 A	74	12	30
	AEB-3 C	7	11	36
	AEB-3D	28	11	17
	AEB-6	3	6	39
Menes-20X	AEB-3 A	7	12	29
	AEB-3 C	15	11	35
	AEB-3D	3	11	42

**Fig. 10:**
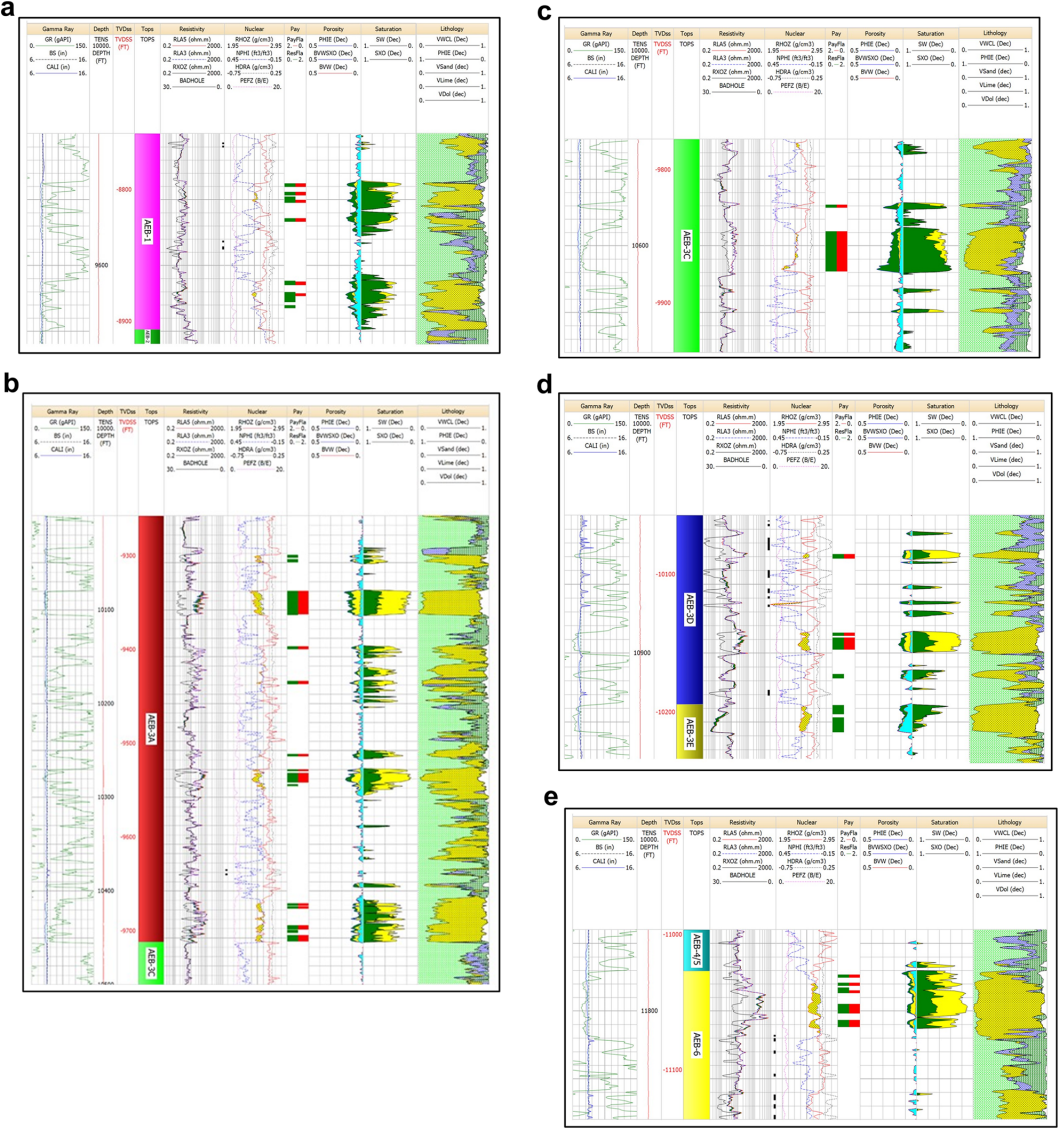
(**a**) Litho-saturation plot for Well Menes-2 in the AEB-1 Unit. The plot shows presence of 14 ft net pay, 11% average porosity and 38% average water saturation. (**b**) Litho-saturation plot for Well Menes-2 in the AEB-3AUnit. The plot shows presence of 60 ft net pay, 12% average porosity and 27% average water saturation. (**c**) Litho-saturation plot for Well Menes-2 in the AEB-3C Unit. The plot shows presence of 32 ft net pay, 13% average porosity and 23% average water saturation. (**d**) Litho-saturation plot for Well Menes-2 in the AEB-3D Unit. The plot shows presence of 14 ft net pay, 10% average porosity and 20% average water saturation. (**e**) Litho-saturation plot for Well Menes-2 in the AEB-6 Unit. The plot shows presence of 16 ft net pay, 8% average porosity and 22% average water saturation.

The petrophysical analysis of Menes-2 well gives positive results in the AEB (1, 3 A, 3 C, 3D & 6) units (Fig. 10a-e). For the AEB-1 unit, the net pay is 14 feet, with an average porosity of 11% and an average water saturation of 38%. The AEB-3 A unit has various zones of cumulative net pay of 60 feet, with 12% average porosity and 27% average water saturation. The AEB-3 C unit shows a net pay of 32 feet, with 13% average porosity and 23% average water saturation. In the AEB-3D unit, the net pay is 14 feet, with 10% average porosity and 20% average water saturation. For AEB-6, the net pay is 16 feet, with 8% average porosity and 22% average water saturation.

**Fig. 11:**
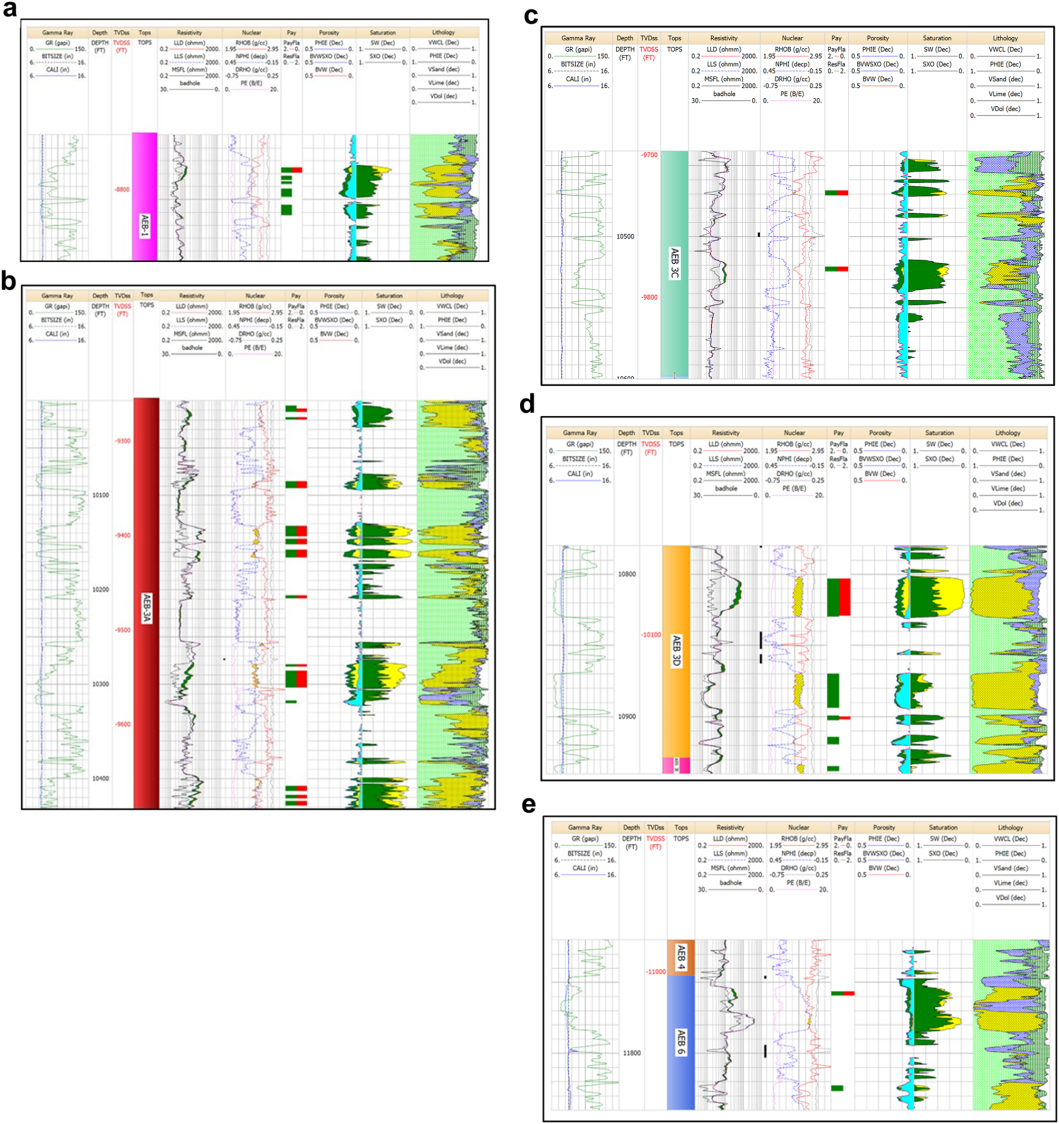
(**a**) Litho-saturation plot for Well Menes-17 in the AEB-1 Unit. The plot shows presence of 4 ft net pay, 10% average porosity and 39% average water saturation. (**b**) Litho-saturation plot for Well Menes-17 in the AEB-3A Unit. The plot shows presence of 74 ft net pay, 12% average porosity and 30% average water saturation. (**c**) Litho-saturation plot for Well Menes-17 in the AEB-3C Unit. The plot shows presence of 7 ft net pay, 11% average porosity and 36% average water saturation. (**d**) Litho-saturation plot for Well Menes-17 in the AEB-3D Unit. The plot shows presence of 28 ft net pay, 11% average porosity and 17% average water saturation. (**e**) Litho-saturation plot for Well Menes-17 in the AEB-6 Unit. The plot shows presence of 3 ft net pay, 6% average porosity and 39% average water saturation.

The promising results in the AEB (1, 3 A, 3 C, 3D & 6) units were obtained after the petrophysical analysis of the Menes-17 (Fig. 11a-e). In the AEB-1 unit, the net pay is 4 feet, with an average porosity of 10% and an average water saturation of 39%. The AEB-3 A unit has multi zones of total net pay of 74 feet, with 12% average porosity and 30% average water saturation. The AEB-3 C unit shows a net pay of 7 feet, with 11% average porosity and 36% average water saturation. In the AEB-3D unit, the net pay is 28 feet, with 11% average porosity and 17% average water saturation. For AEB-6, the net pay is 3 feet, with 6% average porosity and 39% average water saturation.

**Fig. 12:**
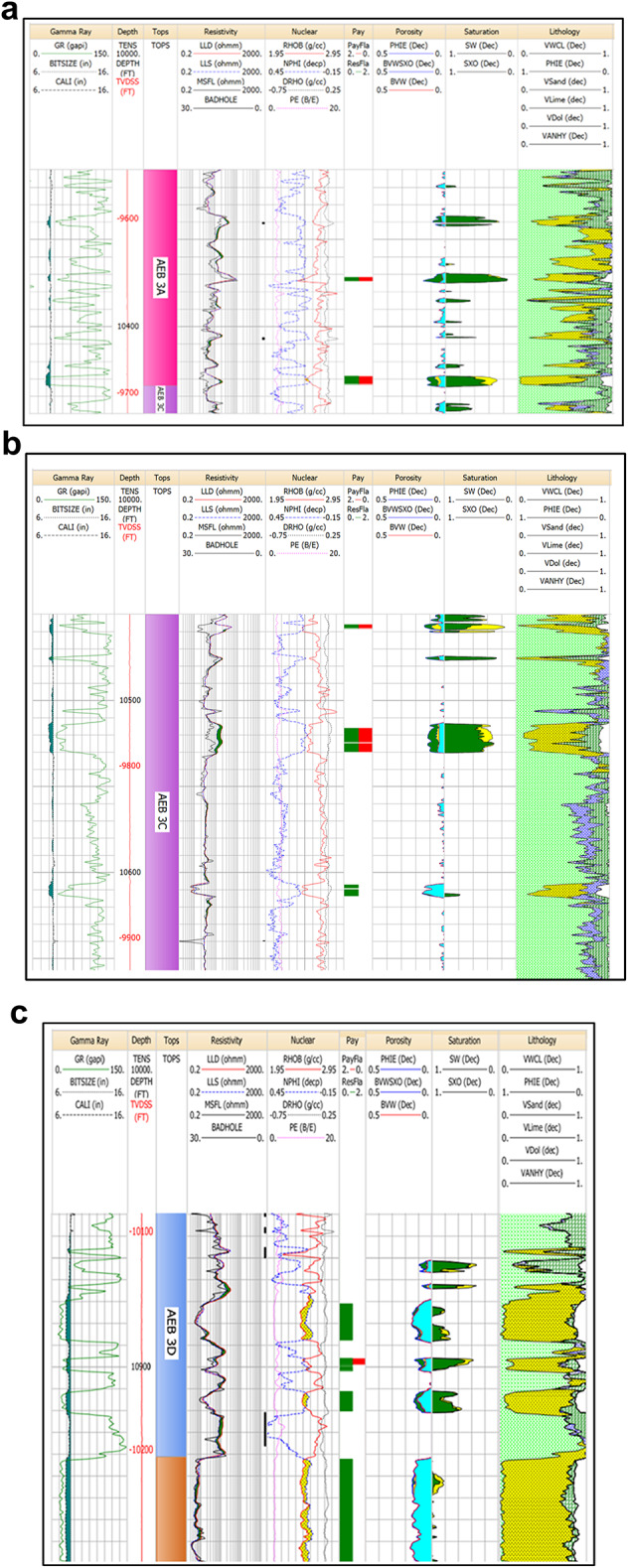
(**a**) Litho-saturation plot for Well Menes-20X in the AEB-3A Unit. The plot shows presence of 7 ft net pay, 12% average porosity and 29% average water saturation. (**b**) Litho-saturation plot for Well Menes-20X in the AEB-3C Unit. The plot shows presence of 15 ft net pay, 11% average porosity and 35% average water saturation. (**c**) Litho-saturation plot for Well Menes-20X in the AEB-3D Unit. The plot shows presence of 3 ft net pay, 11% average porosity and 42% average water saturation.

The petrophysical analysis of Menes-20X shows that pay is present in the AEB (3 A, 3 C & 3D) units (Figs. 12a-c). The AEB-3 A unit has a net pay of 7 feet, with an average porosity of 12% and an average water saturation of 29%. The AEB-3 C unit reveals a net pay of 15 feet, with 11% average porosity and 35% average water saturation. For the AEB-3D unit, the net pay is 3 feet, with 11% average porosity and 42% average water saturation.

Based on the previous results, The following observations were noted:

AEB-3 A is the most laterally continuous reservoir zone in the field, with consistent net pay thickness ranging from 7 ft in Menes-20X to 75 ft in Menes-1X, and an average porosity of approximately 12%. Water saturation varies from 24% in Menes-1X to 30% in Menes-17, indicating differences in charge of hydrocarbon.

Zones AEB-1 and AEB-6 exhibit heterogeneity in both porosity and water saturation.

The structural setting, specifically proximity to faults, expected plays a role in controlling fluid distribution and reservoir quality. For example, Menes-2, located closest to a major normal fault as interpreted from seismic data, displays higher porosity, reaching 13% in AEB-3 C and lower water saturation (20% in AEB-3D to 38% in AEB-1). These characteristics suggest that the fault may have enhanced hydrocarbon charge into the reservoir. Conversely, well Menes-20X, located farther from the major fault, encountered three reservoirs displaying relatively poor petrophysical quality, with high water saturation ranging from 29% in AEB-3 A to 42% in AEB-3D.

### 3D modeling

Integrating multiscale datasets, such as seismic data, which provides a regional view of subsurface structures, with well log data, which offers high-resolution, localized information, provides the following benefits:

Accurate delineate the architectural framework of the Lower Cretaceous reservoir and provide detailed insights into its quality and characterization.

recognize heterogeneities within the reservoir including change in porosity, lithology, and fluid distribution.

reduces uncertainty and increase trust in the geological model^41–43^.

**Fig. 13:**
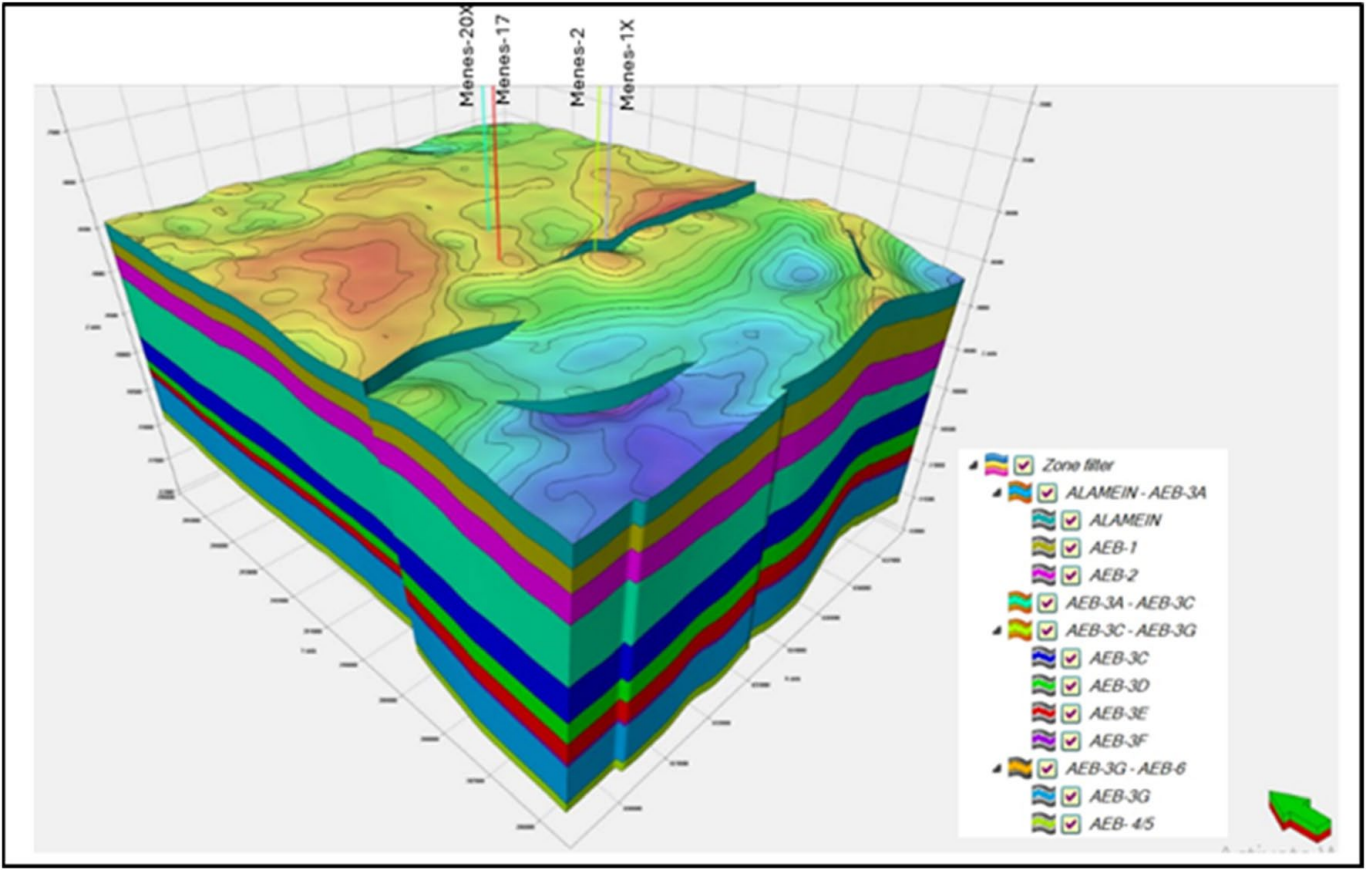
3D depth structural model of the Menes field showing, faults geometry, surfaces, zones, and layers. The model combines interpretation of seismic and well log data to provide a picture for structural features in three dimensions. .

The workflow of modeling is comprised of three main parts: structural, petrophysical and facies modeling^44^. Structural modeling is important for constructing a 3D geological model, as it assists correctly characterizing the geological features and their relationships within the model. The process of building structural models includes three main steps: defining geometry, fault and horizons modeling^5,12,27,45^. The first step, the extension of the required area is automatically delineated using seismic survey data to define the geometric properties. The second step involves modeling faults based on interpreted faults in the depth domain. Then, 2D Grid (Pillar Gridding step) was built via PetrelTM (version 2017.4). Horizon modeling is the last step, and it is achieved by using depth horizon maps for tops of Alamein Formation and tops of Alam El Bueib units (AEB-1 AEB-3 A, AEB-3 C and 3D). Additionally, thickness maps and formation tops data were used to create the other zones and insert into the model conformably with the previous horizons. The created zones are AEB- 2, AEB-3E, AEB-3 F, AEB-3G, AEB-4/5 and AEB-6. The zones were separated into layers of equal thickness. Figure 13 illustrates the created 3D structural model for the present study.

It is noticed that the horizons of Alam El Bueib’s units (1, 3 A, 3 C, and 3D) generally dip towards the south. Also, low relief area is seen in the southern part of this study, while the middle and western parts have highest relief.

**Fig. 14:**
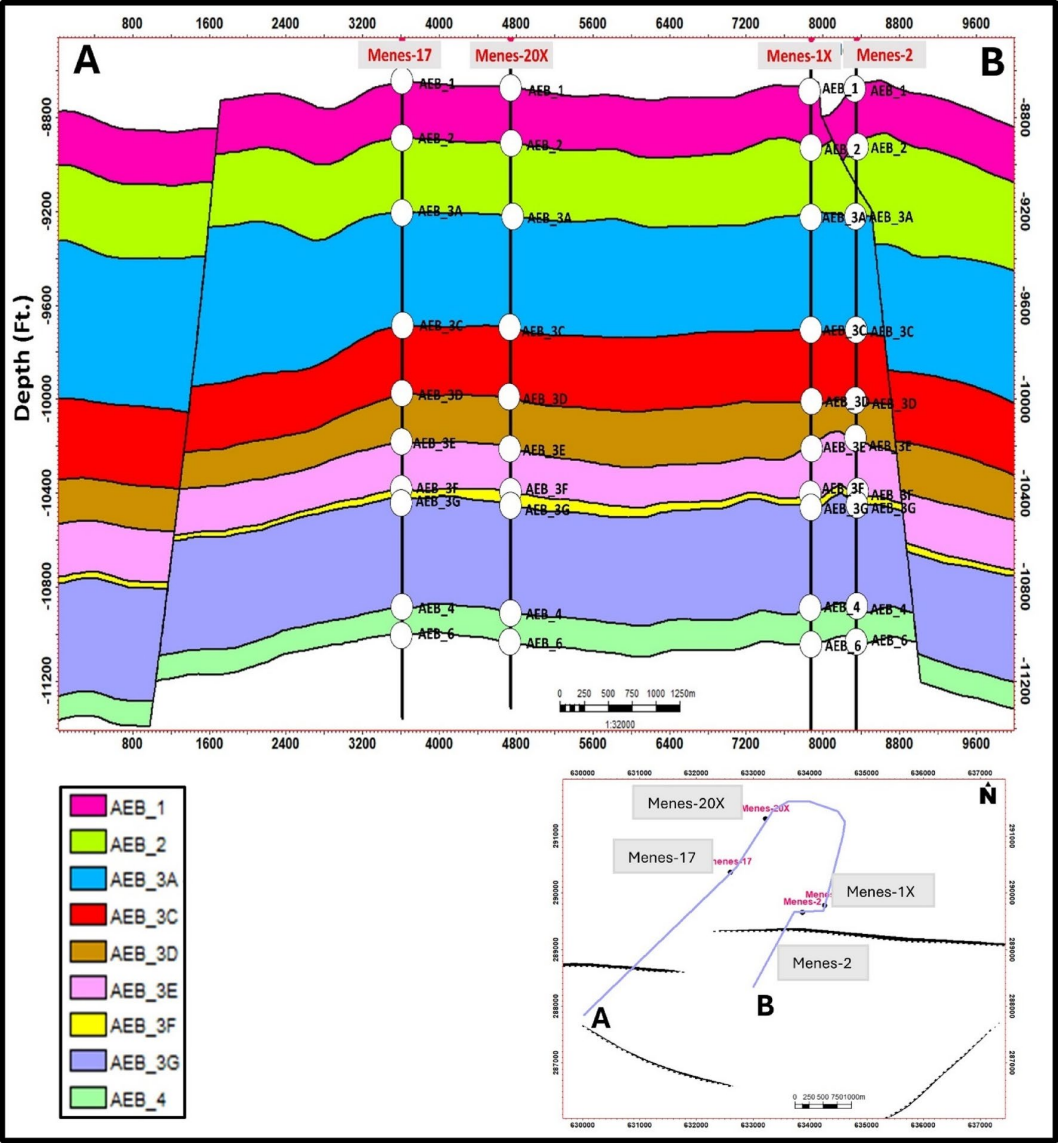
Integrated cross section (A–B) passing through wells Menes-17, Menes-20X, Menes-1X & Menes-2. The section displays lateral variations in reservoir thickness, showing that the area is bound by a major normal fault and the wells were drilled on the up-thrown side.

The cross-section (A-B) passing through Menes wells (Fig. 14) reveals that the three-dimensional structural model has been validated by comparing the modeled horizons with well tops from existing wells to ensure that the model accurately reflects the identified formation depths and structural features at each well location. Moreover, the area is bound by a major normal fault and the wells were drilled on the up-thrown side of a normal fault with (E-W) direction.

### Modeling uncertainties and limitation

The building of the 3D geological model is influenced by the following main factors:

The limited vertical and lateral resolution of seismic data increases uncertainty in identifying horizons and faults. Seismic resolution below AEB-3D may need to be improved through advanced reprocessing techniques or by acquiring new seismic data with updated acquisition parameters^46^.

The limited wells number in the study area decreases confidence in seismic-to-well ties. To reduce this uncertainty, specifically in the southern part of the study area, additional wells with Vertical Seismic Profiling (VSP) data may be required^47^.

Lateral velocity changes, resulting from structural and facies variations, lead to errors in depth estimation, which may affect the accuracy of identifying structural geometry and volumetric calculations^48^.

The structural model is static and lacks geo-mechanical data, such as in-situ stress. Moreover, the absence of dynamic data, like production data and 4D seismic, decreases the ability to validate and predict reservoir performance^49^.

### Implications for future exploration

These uncertainties impact hydrocarbon exploration and production. Incorrect estimation of fault geometry or trap definition may result in drilling dry wells or failing to predict the correct recoverable volumes.

## Conclusion

The key contributions of this study are:

- Understanding the structural configuration in the Menes field. by identifying a series of faults affecting the Lower Cretaceous AEB Formation and defining the key boundaries of the Alamein Dolomite, Alam El Bueib’s units and Paleozoic.
- Recognizing the potential prospects in the Alamein Formation, AEB section, and Paleozoic section, this study presents the up-thrown block in the central part of the study area as the preferred location for drilling new wells.
- Revealing that the structural setting, specifically the proximity to faults, is expected to play a role in controlling fluid distribution and reservoir quality, as illustrated by the Menes-2 well.
- Detecting of a missing stratigraphic section in the Dahab Formation at Menes-2, caused by fault displacement, reflects the effective integration of seismic and well log data for accurate reservoir characterization.
- Suggesting a slight increase in the thickness of the Alam El Buieb section toward the East suggests favorable conditions for sandstone deposition.
- Demonstrating that the Lower Cretaceous Alam El Buieb Formation units (1, 3 A, 3 C, 3D, and 6) are the main hydrocarbon-bearing reservoirs in the study area, and estimating the net pay thickness for each horizon.
- Estimating the key petrophysical parameters like porosity and fluid saturation of the Alam El Buieb reservoirs for wells Menes-1X, Menes-2, Menes-17, and Menes-20X.
- Showing that AEB-3 A is the most laterally continuous reservoir zone in the field, characterized by a consistent net pay thickness.
- Presenting the spatial variations in reservoir quality within the AEB section, likely resulting from the combined effects of structural influences and depositional heterogeneity.

Overall, the construction of the 3D model in the Menes Field provides valuable information that can support strategic decision-making in both exploration and production activities, while also helping to reduce risks associated with drilling and field development planning.

## Data Availability

The data will be available from the corresponding author upon reasonable request and with permission of the Egyptian General Petroleum Cooperation.

## References

[1] EGPC (Egyptian General Petroleum Corporation). Western desert, oil and gas fields. A comprehensive overview. Presented at 11th EGPC petrol. *Explor. Prod. Conf.* (1992).

[2] Cheng, J. E. Petroleum system of Shoushan basin, Western desert, Egypt. *Acta Sci. Malay.* **4** (1), 1–8 (2020).

[3] Sarhan, M. A. & Abdel-Fattah, M. I. Integrating well logs and seismic data for a comprehensive geophysical appraisal of post-Albian oil reservoirs in the SWQ-4X well, Gindi basin, Egypt. *Egypt. J. Pet.* **33** (2), 2 (2024).

[4] Abdel-Fattah, M. & Tawfik, A. 3D geometric modeling of the Abu Madi reservoirs and its implication on the gas development in Baltim Area (Ofshore Nile Delta, Egypt). *Int. J. Geophys.* 11 (2015).

[5] Sarhan, M. A. & Abdel-Fattah, M. I. Geophysical evaluation and petrophysical assessment of the Abu Roash F member: A probable unconventional oil reservoir in Heba field, Eastern Abu gharadig basin, Egypt. *J. Afr. Earth Sci.* **217**, 105330 (2024).

[6] Mahmoud, A. I., Metwally, A. M., Mabrouk, W. M. & Leila, M. Controls on hydrocarbon accumulation in the pre-rift paleozoic and late syn-rift cretaceous sandstones in PTAH oil field, North Western desert, egypt: insights from seismic stratigraphy, petrophysical rock-typing and organic geochemistry. *Mar. Pet. Geol.* **155**, 106398 (2023).

[7] Noureldin, A. M., Mabrouk, W. M. & Metwally, A. Delineating tidal channel feature using integrated post-stack seismic inversion and spectral decomposition applications of the upper cretaceous reservoir Abu Roash C: A case study from Abu-Sennan oil field, Western desert, Egypt. *J. Afr. Earth Sci.* **205**, 104974 (2023).

[8] Noureldin, M., Mabrouk, M. & Metwally, A. Structural and petroleum system analysis using seismic data interpretation techniques to upper cretaceous rock units: A case study, West Abu-Sennan oil field, Northern Western desert, Egypt. *J. Afr. Earth Sci.* **198**, 104772 (2023).

[9] Noureldin, A. M., Mabrouk, W. M. & Metwally, A. M. Superimposed structure in the Southern periphery of Abu gharadig basin, egypt: implication to petroleum system. *Contrib. Geophys. Geodesy*. **53** (2), 97–110 (2023).

[10] Noureldin, M., Mabrouk, W. M., Chikiban, B. & Metwally, A. Formation evaluation utilizing a new petrophysical automation tool and subsurface mapping of the upper cretaceous carbonate reservoirs, the Southern periphery of the Abu-Gharadig basin, Western desert, Egypt. *J. Afr. Earth Sci.* **205**, 104977 (2023).

[11] Mohamed, H., Mabrouk, W. M. & Metwally, A. Delineation of the reservoir petrophysical parameters from well logs validated by the core samples case study sitra field, Western desert, Egypt. *Sci. Rep.* **14** (1), 26841 (2024). 39500975 10.1038/s41598-024-77371-0 PMC11538348

[12] Eid, A. M., Mabrouk, W. M., Amer, M. & Metwally A. 3D structural modeling using seismic data and well logs for Khatatba reservoir in Matruh-Shushan basin, North Western desert, Egypt. *Sci. Rep.* **13** (1), 20158 (2023). 37978307 10.1038/s41598-023-47487-w PMC10656560

[13] Eid, A. M., Mabrouk, W. M. & Metwally, A. Formation evaluation and petrophysical analysis of well logging data: an example from Matruh-Shushan basin, North Western desert, Egypt. *Iraqi J. Sci.* 4336–4358 (2024).

[14] Barakat, M. K. & Nooh, A. Z. Reservoir quality using the routine core analysis data of Abu Roash C in Badr El Din-15 oil field, Abu gharadig basin, North Western desert, Egypt. *J. Afr. Earth Sci.* **129**, 683–691 (2017).

[15] Sultan, N. & Halim, M. A. Tectonic framework of northern Western Desert, Egypt and its effect on hydrocarbon accumulations. In proceedings of the EGPC 9th exploration and production conference, Cairo. *EGPC Bull.*** 2**, 1–19 (1988).

[16] Moustafa, A. R. Mesozoic-Cenozoic basin evolution in the Northern Western desert of Egypt. *Geol. East. Libya*. **3**, 29–46 (2008).

[17] El Awdan, A., Youssef, F. & Moustafa, A. R. Effect of mesozoic and tertiary deformations on hydrocarbon exploration in the Northern Western desert, Egypt (*Am. Assoc. Pet. Geol Int. Meet.* (2002).

[18] Metwalli, F. I., Shendi, E. A. H. & Fagelnour, M. S. Core and well logs interpretation for better reservoir characterization in Shushan basin, Egypt. *Arab. J. Geosci.* **14**, 1–14 (2021).

[19] El-Sayed, A. S., Mabrouk, W. M. & Metwally, A. M. Utilizing post-stack seismic inversion for delineation of gas-bearing sand in a pleistocene reservoir, Baltim gas field, nile delta, Egypt. *Sci. Rep.* **14** (1), 29596 (2024). 39609520 10.1038/s41598-024-78186-9 PMC11605053

[20] El-Sayed, A. S., Mabrouk, W. M. & Metwally, A. M. Pre-stack seismic inversion for reservoir characterization in pleistocene to pliocene channels, Baltim gas field, nile delta, Egypt. *Sci. Rep.* **15** (1), 1180 (2025). 39774632 10.1038/s41598-024-75015-x PMC11706975

[21] Noureldin et al. Hydrocarbon potential in the Northern Egyptian red sea: insights from geophysical datasets and analysis of onshore marginal outcrop analogues and subsurface sequences. *Sci. Rep.* **15** (1), 1198 (2025). 39775084 10.1038/s41598-024-79605-7 PMC11707021

[22] Alotaibi, N. & Metwally, A. Delineation of subsurface structures using seismic refraction tomographic inversion in Wadi Al-Dawasir, South Saudi Arabia. *Eng. Technol. Appl. Sci. Res.***.14** (5), 16519–16526 (2024).

[23] Abuzaied, M., Metwally, A., Mabrouk, W., Khalil, M. & Bakr, A. Seismic interpretation for the jurassic/paleozoic reservoirs of QASR gas field, Shushan-Matruh basin North Western desert, Egypt. *Egypt. J. Pet.* **28** (1), 103–110 (2019).

[24] Abuzaied, M., Mabrouk, W. M., Metwally, A. M., Bakr, A. & Eldin, S. E. Correlation of the reservoir characteristics from the well-logging data and core measurements in QASR field, North Western desert Egypt. *Arab. J. Geosci.* **13**, 1–9 (2020).

[25] Abdel-Fattah, M. I., Metwalli, F. I. & El Sayed, I. M. Static reservoir modeling of the Bahariya reservoirs for the oilfields development in South Umbarka area, Western desert, Egypt. *J. Afr. Earth Sci.* **138**, 1–13 (2018).

[26] Metwalli, F. I., Shendi, E. A. H. & Fagelnour, M. S. Seismic facies analysis of thin sandstone reservoirs, North Western desert, Egypt. *J. Pet. Explor. Prod. Technol.* **9**, 793–808 (2019).

[27] Lasheen, I., Noureldin, A. M. & Metwally, A. Reservoir characterization of the Abu Roash D member through petrography and seismic interpretations in Southern Abu gharadig basin, Northern Western desert, Egypt. *Sci. Rep.* **14** (1), 8966 (2024). 38637582 10.1038/s41598-024-58846-6 PMC11026448

[28] Amer, M., Mabrouk, W. M., Soliman, S., Noureldin, M. & Metwally, A. Tree-dimensional integrated geo-static modeling for prospect identification and reserve Estimation in the middle miocene multi-reservoirs: A case study from Amal field, Southern Gulf of Suez Province. *Nat. Resour. Res.* **32** (6), 2609–2635 (2023).

[29] Al-Chalabi, M. Time-depth relationships for multilayer depth conversion. *Geophys. Prospect.* **45**, 715–720 (1997).

[30] Sherrif, R. E.* Encyclopedic Dictionary of Exploration Geophysics*. 3rd Edition. SEG (1991).

[31] Fagelnour, M. S., Metwalli, F. I. & Shendi, E. A. H. Structural and facies modeling of the lower cretaceous Alam El Bueib reservoirs in the Shushan basin, Western desert, Egypt. *Arab. J. Geosci.* **11** (18), 553 (2018).

[32] Amosu, A., Sun, Y. & MinInversion A program for petrophysical composition analysis of geophysical well log data. *Geosciences* **8** (2), 65 (2018).

[33] Amer, M., Mabrouk, W. M., Eid, A. M. & Metwally, A. Petrophysical assessment of the Hammam faraun, Matulla and Nubia reservoirs in the Ashrafi oil field, Gulf of Suez. *Sci. Rep.* **15** (1), 3326 (2025). 39865084 10.1038/s41598-025-86297-0 PMC11770147

[34] Metwally, M. et al. Formation evaluation of Abu Madi reservoir in Baltim gas field, nile delta, using well logs, core analysis, and pressure data. *Sci. Rep.* **13** (1), 19139 (2023). 37932367 10.1038/s41598-023-46039-6 PMC10628261

[35] Chikiban, B., Kamel, M. H., Mabrouk, M. & Metwally, A. M. Petrophysical characterization and formation evaluation of sandstone reservoir: case study from Shahd field, Western desert, Egypt. *Contrib. Geophys. Geodesy*. **52** (03), 443–466 (2022).

[36] Hassan, M., Mabrouk, W. M. & Farhoud, M. Petrophysical analysis for Ammonite-1 well, Farafra area, Western desert, Egypt. *Arab. J. Geosci.* **7** (12), 5107–5125 (2014).

[37] Asquith, G. B. & Gibson, C. R. *Basic Well Log Analysis for Geologists: American Association of Petroleum Geologists* Tulsa, 216. (1982).

[38] Stephens, D. B. et al. A comparison of estimated and calculated effective porosity. *Hydrogeol. J.* **6**, 156–165 (1998).

[39] Amer, M., Mabrouk, W. M., Soliman, K. S., Eid, A. M. & Metwally, A. Formation evaluation of middle miocene reservoirs via petrophysical analysis of well logging data; case study from Southern part of Gulf of suez, Egypt. *Iran. J. Geophys.* **18** (3), 39–57 (2024).

[40] Metwally, A. M., Mabrouk, M. & Mahmoud, I. A numerical approach to accurately estimate water resistivity (Rw) and saturation (Sw) in Shaly sand formations. *Contrib. Geophys. Geodesy*. **52** (3), 423–441 (2022).

[41] Sarhan, M. A., Shehata, A. A. & Abdel-Fattah, M. I. Sequence stratigraphic and petrophysical controls on the oil-reservoirs architecture: A case study from the cretaceous meqasequence, Gulf of Suez region, Egypt. *J. Afr. Earth Sci.* **219**, 105412 (2024).

[42] Turner, R. et al. Structural and stratigraphic controls on reservoir architecture: A case study from the lower oligocene Vicksburg formation, Brooks county, Texas. *Mar. Pet. Geol.* **160**, 106627 (2024).

[43] Shehata, A. A. et al. Sequence stratigraphy and petrophysical evaluation controls on fluvial to fluvio-marine reservoirs in the syn-and post-rift sediments: insights into in the Kharita and Bahariya formations, West Beni suef basin. *J. Afr. Earth Sci.* **226**, 105568 (2025).

[44] Abdel-Fattah, M., Hanafy, M., Hamdan, H. & Attia, T. Integrated three dimensional reservoir modeling and tectonic evaluation of the upper cretaceous Bahariya formation in the burg El Arab area, egypt: an implications for hydrocarbon exploration and production strategies. *Egypt. J. Pet.* **33** (2), 259–268 (2024).

[45] Alcalde, J. et al. The importance of structural model availability on seismic interpretation. *J. Struct. Geol.* **97**, 161–171 (2017).

[46] Faleide, T. S. et al. Impacts of seismic resolution on fault interpretation: insights from seismic modelling. *Tectonophysics* **816**, 229008 (2021).

[47] Ahmed, J. Z., Fatah, A. A., Malla, L., Blooshi, H. A. & Maskari, S. A. Characterizing Carbonate Reservoir to Capture the Details of the Vertical and Spatial Heterogeneity Using an Integrated Workflow: A Case Study from Onshore Abu Dhabi. *In Abu Dhabi International Petroleum Exhibition and Conference*, D041S156R005 (2024).

[48] Totake, Y., Butler, R. W. & Bond, C. E. Structural validation as an input into seismic depth conversion to decrease assigned structural uncertainty. *J. Struct. Geol.* **95**, 32–47 (2017).

[49] Molenaar, M. M. et al. Applying Geo-Mechanics and 4D: 4D In-Situ Stress as a Complementary Tool for Optimizing Field Management. In *ARMA North America Rock Mechanics Symposium* ARMA (2004).

[50] Abd-Allah, Z. M., Maky, A. F. & Ramadan, M. A. Organic source of crude oils and 1D basin modeling of upper cretaceous rocks, Badr concession, Abu gharadig basin, Western desert, Egypt. *Arab. J. Geosci.* **11**, 1–13 (2018).

